# Cardiac magnetic resonance imaging in the German National Cohort (NAKO): Automated segmentation of short-axis cine images and post-processing quality control

**DOI:** 10.1016/j.jocmr.2025.101958

**Published:** 2025-09-12

**Authors:** Peter M. Full, Robin T. Schirrmeister, Manuel Hein, Maximilian F. Russe, Marco Reisert, Clemens Ammann, Karin Halina Greiser, Thoralf Niendorf, Tobias Pischon, Jeanette Schulz-Menger, Klaus H. Maier-Hein, Fabian Bamberg, Susanne Rospleszcz, Christopher L. Schlett, Christopher Schuppert

**Affiliations:** a Division of Medical Image Computing, German Cancer Research Center (DKFZ), Heidelberg, Germany; b Medical Faculty Heidelberg, Heidelberg University, Heidelberg, Germany; c Medical Physics, Department of Diagnostic and Interventional Radiology, Medical Center – University of Freiburg, Faculty of Medicine, University of Freiburg, Freiburg, Germany; d Department of Cardiology and Angiology, University Heart Center Freiburg – Bad Krozingen, Medical Center – University of Freiburg, Faculty of Medicine, University of Freiburg, Freiburg, Germany; e Department of Diagnostic and Interventional Radiology, Medical Center – University of Freiburg, Faculty of Medicine, University of Freiburg, Freiburg, Germany; f Charité – Universitätsmedizin Berlin, Corporate Member of Freie Universität Berlin and Humboldt-Universität zu Berlin, Berlin, Germany; g Working Group on Cardiovascular Magnetic Resonance, Experimental and Clinical Research Center (ECRC), a joint cooperation between the Charité – Universitätsmedizin Berlin and the Max-Delbrück-Center for Molecular Medicine, Berlin, Germany; h German Centre for Cardiovascular Research (DZHK), Partner Site Berlin, Berlin, Germany; i Department of Cardiology and Nephrology, HELIOS Hospital Berlin-Buch, Berlin, Germany; j Division of Cancer Epidemiology, German Cancer Research Center (DKFZ), Heidelberg, Germany; k Berlin Ultrahigh Field Facility (B.U.F.F.), Max Delbrück Center for Molecular Medicine in the Helmholtz Association (MDC), Berlin, Germany; l Max-Delbrück-Center for Molecular Medicine in the Helmholtz Association (MDC), Molecular Epidemiology Research Group, Berlin, Germany; m Max-Delbrück-Center for Molecular Medicine in the Helmholtz Association (MDC), Biobank Technology Platform, Berlin, Germany

**Keywords:** Cardiac MR imaging, Population imaging, Artificial intelligence, Quality control, German National Cohort

## Abstract

**Background:**

The prospective, multicenter German National Cohort (NAKO) provides a unique dataset of cardiac magnetic resonance (CMR) cine images. Effective processing of these images requires a robust segmentation and quality control pipeline.

**Methods:**

A deep learning model for semantic segmentation, based on the nnU-Net architecture, was applied to full-cycle short-axis cine images from 29,908 baseline participants. The primary objective was to determine data on structure and function for both ventricles (LV, RV), including end-diastolic volumes, end-systolic volumes, and LV myocardial mass. Statistical and visual quality control was performed, including an expert assessment of outliers in morphofunctional parameters, inter- and intra-ventricular phase differences, and LV time-volume curves (TVC). These were adjudicated using a five-point rating scale, ranging from five (excellent) to one (non-diagnostic), with ratings of three or lower subject to exclusion. The predictive value of outlier criteria for inclusion and exclusion was evaluated using receiver operating characteristics analysis.

**Results:**

The segmentation model generated complete data for 29,609 of 29,908 participants (99.0%), of whom 5082 (17.0%) underwent visual assessment. Quality assurance yielded a final sample of 26,899 (89.9%) participants with excellent or good quality, excluding 1875 participants due to image quality issues and 835 participants due to segmentation quality issues. TVC was the strongest single discriminator between included and excluded participants (AUC: 0.684). Of the two-category combinations, the pairing of TVC and phases provided the greatest improvement over TVC alone (AUC difference: 0.044; p<0.001). The best performance was observed when all three categories were combined (AUC: 0.748). By extending the quality-controlled sample to include mid-level “acceptable” quality ratings, a total of 28,413 (95.0%) participants could be included.

**Conclusion:**

The implemented pipeline enabled automated segmentation of an extensive CMR dataset and integrated thorough quality control measures, providing a comprehensive and reliable data resource for quantitative analyses with diminished risk of bias.

## Graphical abstract

**Figure:**
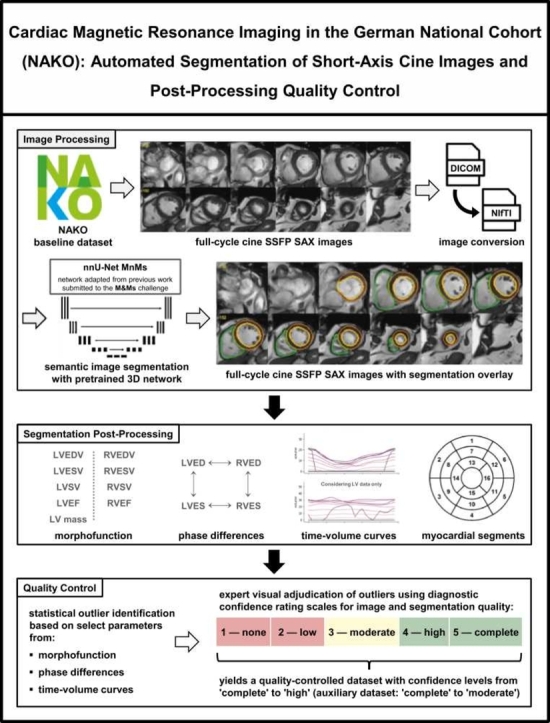

## 1 Background

Cardiac magnetic resonance imaging (CMR) is widely acknowledged as the standard for the noninvasive evaluation of cardiac structure and function. Its ability to provide detailed quantification of ventricular volumes, myocardial mass, and functional parameters has made it indispensable in both clinical practice and cardiovascular research [1], [2]. Extraction of these parameters is typically performed on contiguous stacks of cine images acquired in the short-axis orientation. For large-scale cohorts, such as those encountered in population imaging studies aimed at understanding the epidemiology of cardiovascular diseases, conventional—manual or semi-manual—segmentation methods for short-axis cine images are deemed impractical, primarily due to their labor-intensive nature and susceptibility to inter- and intra-observer variability. Moreover, suboptimal reproducibility across multicenter datasets may mask subtle variations in cardiac morphology and function across diverse populations. This issue is additionally complicated by variations in image quality, which may be adversely affected by diverse factors, including image artifacts associated with breathing or cardiac motion when ineffectively compensated for by electrocardiogram (ECG) gating. Overcoming these challenges demands advanced automated segmentation approaches and thorough quality control measures.

Automated segmentation techniques for CMR cine images have recently emerged through innovations in deep learning, notably the nnU-Net architecture [3], [4]. These approaches now generate segmentation data with high robustness and achieve accuracies comparable to inter-observer variability on an average level, while helping to reduce biases [5], [6]. Nonetheless, their use in large population studies still requires thorough quality control [7], [8]. When implemented effectively, their application in population imaging and epidemiology has enabled the creation of reliable CMR datasets that support the public health objective of disease prevention [9], [10].

The present study adopts methods from the first-ranked contribution to a recent segmentation challenge [5], [6] and data from the prospective, multicenter German National Cohort (NAKO) study to develop an image processing pipeline specifically tailored for short-axis cine CMR images. By integrating a deep learning model for automated segmentation with a comprehensive post-processing quality control framework, it aims to generate a quality-assured dataset of comprehensive CMR parameters that enables downstream analyses and ensures their integrity.

## 2 Methods

### 2.1 Ethics and participant consent

The NAKO Use and Access Committee approved this project based on participants’ informed consent, its accordance with the aims of the NAKO, and ethical approval from the Ethics Committee of the Medical Faculty of Heidelberg University, Germany (S-972/2020, approved on February 3, 2021). This study conformed to the ethical guidelines of the 1964 Declaration of Helsinki and its amendments.

### 2.2 Project design

**Fig. 1:**
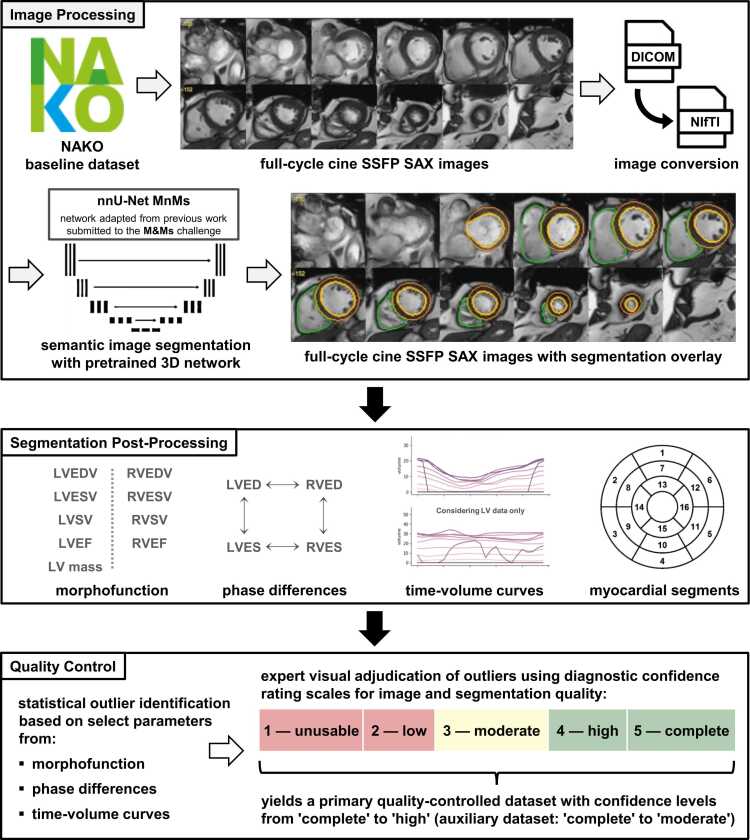
Overview of the study design, including the developed pipeline for image segmentation and quality control. *ACDC* Automated Cardiac Diagnosis Challenge, *DICOM* Digital Imaging and Communications in Medicine, *LV* left ventricle, *LVED* left ventricular end diastole, *LVEDV* left ventricular end diastolic volume, *LVEF* left ventricular ejection fraction, *LVES* left ventricular end systole, *LVESV* left ventricular end systolic volume, *LVSV* left ventricular stroke volume, *M&Ms* Multi-Centre, Multi-Vendor and Multi-Disease Cardiac Segmentation Challenge, *NIfTI* Neuroimaging Informatics Technology Initiative, *RVED* right ventricular end diastole, *RVEDV* right ventricular end diastolic volume, *RVEF* right ventricular ejection fraction, *RVES* right ventricular end systole, *RVESV* right ventricular end systolic volume, *RVSV* right ventricular stroke volume, *SAX* short-axis, *SSFP* steady-state free precession

An overview of the design is provided in Fig. 1.

Our project was built on data from the NAKO, which is an ongoing, prospective, multicenter, population-based cohort study conducted by a network of 25 institutions across 18 regional examination sites in Germany. Its main objective is to investigate risk factors for the development of common chronic diseases such as cancer, diabetes, cardiovascular, neurodegenerative/psychiatric, respiratory, and infectious diseases [11]. The baseline assessment was conducted between 2014 and 2019, enrolling 205,415 participants from the general population aged 19 to 74 years independently of known cardiovascular disease. The embedded NAKO MR imaging study enrolled 30,868 of these participants to undergo whole-body MR examination [12].

A detailed technical description of whole-body MR imaging in the NAKO has been published previously [13]. MR imaging was performed at five dedicated imaging centers using identical 3T whole-body MR scanners (MAGNETOM Skyra, Siemens Healthineers, Erlangen, Germany) running the same software version. All sites adhered to a standardized imaging protocol that included a cardiac assessment through functional and quantitative imaging techniques, incorporating the acquisition of a steady-state free precession (SSFP) full-cycle cine image stack in the short-axis (SAX) orientation. By default, this consisted of 12 slices with 6 mm section thickness (10 mm spacing between slices), covering the heart from base to apex, with 25 phases evenly distributed over the cardiac cycle [13]. All image acquisitions were performed according to a standard operating procedure by radiologic technologists specifically trained and certified for the study. Cardiac planes were automatically planned using vendor-provided software (Cardiac Dot Engine, Siemens Healthineers, Erlangen, Germany). The technologists were instructed to repeat any protocol if anatomic coverage did not meet the SOP, if severe image artifacts occurred, or if image quality was unsatisfactory for other reasons [14].

For our project, we considered all available data at the time of investigation, comprising baseline whole-body MR examinations from 30,868 participants (excluding data from participants who withdrew consent).

### 2.3 Image processing

Short-axis cine images were available for 29,908 participants. These images were converted from the DICOM (Digital Imaging and Communications in Medicine) format to the NIfTI (Neuroimaging Informatics Technology Initiative) format using an open-source tool [15]. They were then processed using a deep learning-based segmentation algorithm built on the nnU-Net framework, which had been fine-tuned for CMR image data during the M&Ms challenge and was the first-ranked contribution by members of the present author group based on excellent accuracy and generalizability to data from previously unseen scanner vendors and imaging sites [5], [16]. As part of the published segmentation challenge, the models were trained on a public dataset annotated by experts following the guidelines of the Society for Cardiovascular Magnetic Resonance [17]. The model predicts each frame of the cardiac cycle using an ensemble comprising five 2D and five 3D segmentation networks. Further details on training and model configuration are provided in a separate publication [6]. The algorithm output comprises full-cycle annotations for the endocardial and epicardial contours of the LV (together forming the myocardial segmentation) as well as the endocardial contour of the RV.

**Fig. 2:**
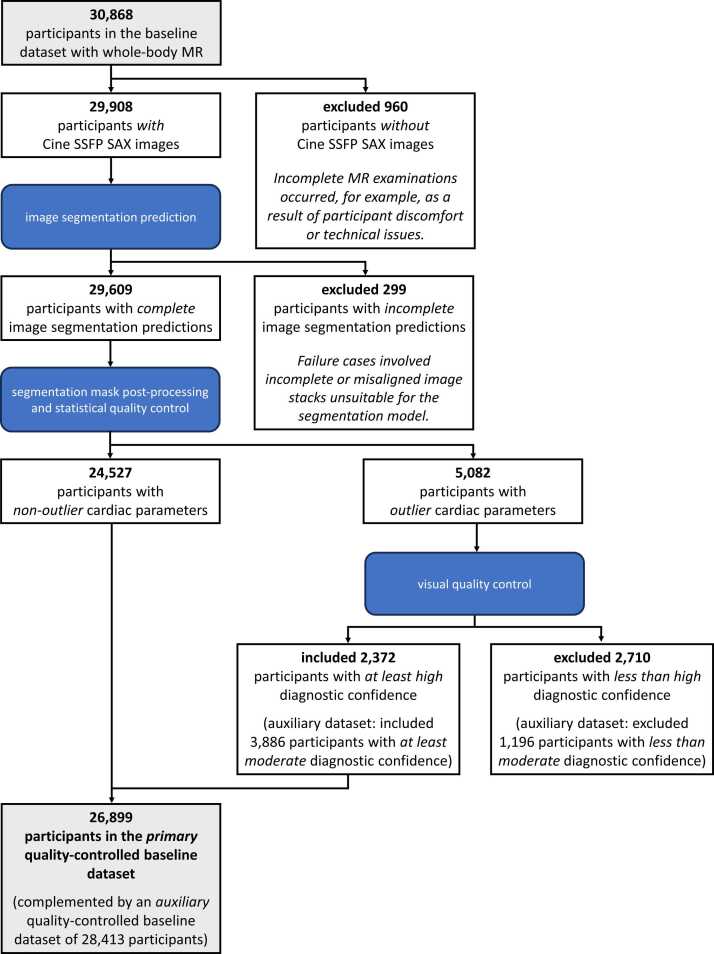
Flowchart of the study sample. The upper half illustrates data described in the Methods section, whereas the lower half presents data from the Results section. *MR* magnetic resonance, *SAX* short-axis, *SSFP* steady-state free precession

To assess the extent of variation between different annotation methods in quantitative results, we initially applied the segmentation algorithm to a limited “data freeze” dataset comprising cine SSFP SAX images from an early subset of 11,050 NAKO baseline participants, made available prior to the release of the full baseline MR imaging dataset. A random sample of 30 participants was drawn and additionally processed in two software environments widely established for CMR images analysis: syngo.via (Siemens Healthineers, Erlangen, Germany) and cvi42 (Circle Cardiovascular Imaging, Calgary, Canada). The contours automatically generated by these systems were manually reviewed and corrected by two expert annotators (syngo.via: M.H., 9 years of experience; cvi42: C.S., 4 years of experience). A comparison demonstrated strong agreement based on Dice similarity coefficients with mean values for the LV cavity, LV myocardium, and RV cavity ranging between 0.88 and 0.95 **(Supplemental Table S1)**. It also showed that variabilities between morphofunctional parameters from the segmentation algorithm and either expert assessment did not exceed those between the two expert assessments **(Supplemental Figure S2)**. Therefore, following finalization of the quality control methodology (detailed in the “Quality control” section below), we used the unmodified version of this validated algorithm for the present research project and applied it to the full baseline dataset, containing cine SSFP SAX images from 29,908 participants. This process generated complete prediction data for 29,609 participants, while 299 participants (1.0%) had incomplete data due to missing segmentations for one or more phases **(**Fig. 2**)**. The failure cases involved image stacks that were largely incomplete or severely misaligned, placing them outside the scope of the segmentation model.

### 2.4 Segmentation post-processing

The output of the segmentation algorithm was utilized to identify the end-diastolic and end-systolic phases for both ventricles based on maximum and minimum volumes (t_LVED_, t_LVES_, t_RVED_, and t_RVES_). These cardiac phases were subsequently used to calculate inter-ventricular phase differences at end-diastole (dp_LVED/RVED_) and end-systole (dp_LVES/RVES_), as well as intra-ventricular phase differences between end-diastole and end-systole (dp_LVED/LVES_, dp_RVED/RVES_). Additionally, the following morphofunctional parameters were derived: ventricular end-diastolic and end-systolic volumes (LVEDV and LVESV, RVEDV, and RVESV), ventricular stroke volumes (LVSV, RVSV), ventricular ejection fractions (LVEF, RVEF), and left-ventricular myocardial mass (LV mass, calculated at end-diastole using a tissue density of 1.055 g/mL).

Using a combination of temporal data and segmentation data, time-volume curves (TVC) for the LV blood pool were computed and analyzed: Principal Component Analysis was applied to a matrix of dimensions n × 25 (with n representing the number of participants, 25 representing phases) to identify the dominant modes of variation in LV volume dynamics. Each mode captured progressively less variance (e.g., Mode 0 corresponds to the highest variance, Mode 1 to the second highest, etc). This approach assigned distinctive numerical values to each temporal evolution of the volume, thereby enabling quantitative analyses such as outlier detection. Simultaneously, it allowed visualization and verification of the plausibility of the identified temporal volume modes.

By further processing the segmentation data, a 17-segment model for myocardial wall thickness at end-diastole was computed, following recommendations from the American Heart Association (AHA) [18]. Additional methodological details, including a schematic overview **(Supplemental Figure S3)**, are presented in the Supplemental Materials.

### 2.5 Quality control

The results from the limited dataset were assessed to identify participants with outlier values. For the morphofunctional parameters LVEDV, LVESV, LV mass, RVEDV, and RVESV—each with and without normalization to body surface area (BSA)—as well as LVEF and RVEF, the ten participants with the highest and lowest values were included in an exploratory sample for visual review. The expected total of 140 participants was reduced by one due to overlapping outlier values across multiple parameters for the same individual, resulting in a sample of 139 distinct participants. A random selection of 200 participants was added, and two expert readers (P.M.F., 3 years of experience; C.S.) jointly reviewed all 339 participants using an in-house assessment tool, based on the open-source image viewer *napari* [19]. The tool was operated in a dual-layout mode, displaying the short-axis cine images with and without the segmentation overlay. Based on their findings related to the representation of cardiac structures and accuracy of segmentations, detailed criteria were established for two five-point rating scales of diagnostic confidence (5—complete confidence [no relevant flaws], 4—high confidence [minor flaws unlikely to have a substantial effect on parameters], 3—moderate confidence [flaws that may have a non-negligible effect depending on the specific parameter], 2—low confidence [limited information of cardiac morphofunction], 1—unusable [very limited or no usable information of cardiac morphofunction]): A first rating scale for evaluating the image quality, especially considering the presence and severity of image artifacts (including spatial and temporal inconsistencies from breathing or inconsistent ECG synchronization), misalignment of the image stack from the intended short-axis orientation, and missing slices. A second rating scale for evaluating the segmentation quality, focusing on the presence and severity of oversegmentation or undersegmentation errors, including transposition of segmentations into other organs or missing segmentations. Effects typically associated with the segmentation model—such as potential “fraying” between segmentations of adjacent slices at the base or apex, attributable to interpolation or partial-volume effects—were classified as segmentation artifacts and exempted from penalization rather than being designated as segmentation errors. In both rating scales, particular emphasis was placed on aspects relevant to future research applications, supported by clinical reasoning, especially concerning the severity of potentially detrimental findings. The combined rating could not exceed the initial image quality rating. The final rating scales are presented in **Supplemental Figure S4**, along with additional explanatory notes.

The results from the full baseline dataset were subsequently analyzed to identify statistical outliers in three categories: morphofunctional parameters, phase differences, and TVC. For morphofunctional parameters (LVEDV, LVESV, LV mass, RVEDV, and RVESV [all normalized to BSA], as well as LVEF and RVEF), and the inter-ventricular difference in stroke volumes (dSV; calculated as “LVSV minus RVSV”), outlier values were defined as those exceeding or falling below 2.5 standard deviations from the sex-specific median. For phase differences, outliers were defined as those exceeding two cardiac phases in the case of inter-ventricular differences (dp_LVED/RVED_, dp_LVES/RVES_), and as those shorter than six cardiac phases in the case of intra-ventricular differences (dp_LVED/LVES_, dp_RVED/RVES_). For TVC, outliers were identified as participants with first mode scores above the 91st percentile. These outlier definitions were based on a combination of statistical conventions, practical considerations, and physiological reasoning. The identified participants were visually assessed by a single expert reader (C.S.) using a local instance of the NORA imaging platform [20]. A multi-layout view was chosen, displaying the short-axis cine images both with and without the segmentation overlay, alongside long-axis images in two-, three-, and four-chamber views for reference **(Supplemental Figure S5)**. Comparing between multiple imaging planes aided in differentiating between image artifacts and asynchronous cardiac motion patterns. There was no blinding to which parameters identified the participant as an outlier. Based on the final ratings, a primary quality-controlled dataset was constructed by excluding participants who received scores of ≤3 in either image quality or segmentation quality (retaining those rated 4 or 5). For a more permissive alternative, an auxiliary dataset was constructed by excluding participants with scores of ≤2 in either rating (retaining those rated 3, 4, or 5).

### 2.6 Statistics

Participant flows are presented as counts and percentages. Cardiac morphofunctional parameters are presented as continuous values. Agreement between nnU-Net segmentations and those of two expert annotators was assessed in a random sample of 30 participants using Dice similarity coefficients and further visualized with Bland-Altman plots as well as scatter plots, along with Pearson correlation coefficients. Proportions of outliers, image quality ratings, and exclusions are described for the whole sample, and stratified by sex, age groups, and imaging sites. Fitted values from a generalized linear model with logit link were used to construct receiver operating characteristic (ROC) curves, with corresponding areas under the curve (AUCs) calculated for outcome exclusion based on single outlier categories and their combinations. Differences in AUCs were compared using the DeLong test. Differences in median morphofunctional parameter values between included and excluded participants were compared using the Mann–Whitney U test and illustrated with violin plots. p-values less than 0.05 were considered statistically significant. Analyses used SAS version 9.4 (SAS Institute Inc., Cary, North Carolina, USA) and R version 4.4.2 (R Foundation for Statistical Computing, Vienna, Austria).

## 3 Results

### 3.1 Quality control outcomes

**Fig. 3:**
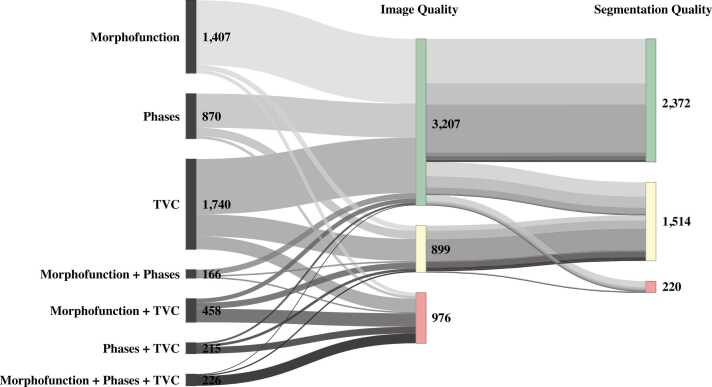
Sankey diagram illustrating multistep quality control outcomes for CMR data from 5082 participants. The first column groups participants into individual and combined outlier categories. The second and third columns display diagnostic confidence ratings for image and segmentation quality, respectively. Confidence levels are color-coded: green indicates complete or high confidence (ratings 5–4), blue indicates moderate confidence (3), and red indicates low or no confidence (2–1). *CMR* cardiac magnetic resonance (imaging), *TVC* time-volume curves

Fig. 3 visualizes the quality control outcomes described below. **Supplemental Figure S6** provides representative examples of quality issues that, depending on severity, prevented inclusion into the quality-controlled datasets.

Based on morphofunctional parameters, cardiac phases, and time-volume curves—along with threshold limits established from these data for statistical quality control—5082 (17.0%) participants with complete segmentation data were identified as outliers. The majority of these were associated with a single outlier category, accounting for 4017 participants (79.0% of all outliers), whereas 839 (16.5%) and 226 (4.4%) participants were associated with two and three outlier categories, respectively. Visual assessment led to acceptance rates ranging from 35.1% to 44.8% per outlier category, and from 8.6% to 69.0% per parameter **(**Table 1**)**. This corresponded to the exclusion of 1875 outliers due to image quality and an additional 835 for segmentation quality issues. Thus, 2710 of 5082 outliers (53.3%) were excluded, leaving 26,899 of 29,908 participants in the *primary* quality-controlled baseline dataset, which included only cases with complete or high diagnostic confidence (inclusion rate: 89.9%, Fig. 2). Among the heterogeneous issues observed, ECG mistriggering and breathing artifacts were particularly common. Key demographic and health-related characteristics, as well as final CMR metrics of this dataset are provided in Table 2.

**Table 1:** Results from the outlier analysis of the processed baseline dataset.

Outlier Category	Parameter	Limit	n identified	n included	
Any			5082	2372	(46.7%)
Morphofunction	Any	outside±2.5 SD of the gender-specific median in the dataset	2257	1011	(44.8%)
	LVEDV /BSA _high_	> 102.3 mL/m^2^ (f) > 115.2 mL/m^2^ (m)	264	176	(66.7%)
	LVEDV /BSA _low_	< 38.1 mL/m^2^ (f) < 34.9 mL/m^2^ (m)	119	59	(49.6%)
	LVESV /BSA _high_	> 40.7 mL/m^2^ (f) > 48.6 mL/m^2^ (m)	471	179	(38.0%)
	LVESV /BSA _low_	< 9.5 mL/m^2^ (f) < 9.3 mL/m^2^ (m)	22	8	(36.4%)
	LVEF _high_	> 79.5% (f) > 77.6% (m)	33	21	(63.6%)
	LVEF _low_	< 48.9% (f) < 45.2% (m)	476	41	(8.6%)
	LV mass /BSA _high_	> 66.9 g/m^2^ (f) > 86.5 g/m^2^ (m)	449	310	(69.0%)
	LV mass /BSA _low_	< 34.5 g/m^2^ (f) < 41.3 g/m^2^ (m)	28	4	(14.3%)
	RVEDV /BSA _high_	> 113.9 mL/m^2^ (f) > 130.2 mL/m^2^ (m)	270	183	(67.8%)
	RVEDV /BSA _low_	< 39.1 mL/m^2^ (f) < 41.4 mL/m^2^ (m)	125	52	(41.6%)
	RVESV /BSA _high_	> 54.7 mL/m^2^ (f) > 65.4 mL/m^2^ (m)	421	159	(37.8%)
	RVESV /BSA _low_	< 10.8 mL/m^2^ (f) < 13.4 mL/m^2^ (m)	20	2	(10.0%)
	RVEF _high_	> 75.8% (f) > 72.8% (m)	93	58	(62.4%)
	RVEF _low_	< 38.7% (f) < 35.3% (m)	321	49	(15.3%)
	dSV _high_	> 36.1 mL/m^2^ (f) > 41.4 mL/m^2^ (m)	300	106	(35.3%)
	dSV _low_	< –31.4 mL/m^2^ (f) < –42.1 mL/m^2^ (m)	164	71	(43.3%)
Phases	Any		1477	518	(35.1%)
	dp_LVED/LVES_	< 6	81	8	(9.9%)
	dp_RVED/RVES_	< 6	77	7	(9.1%)
	dp_LVED/RVED_	> 2	760	265	(34.9%)
	dp_LVES/RVES_	> 2	703	246	(35.0%)
Time-Volume Curves	Mode 1	> 91st percentile	2639	1041	(39.4%)

For each of the parameters above, *n identified* specifies the number of participants identified as outliers, according to threshold limits from statistical quality control that were normalized separately for females (f) and males (m), where applicable. Following expert visual review, *n included* specifies the number of participants in this outlier sample with complete or high diagnostic confidence regarding data quality (ratings 5–4), along with the corresponding percentage relative to *n*. Since participants may exhibit outlier values across multiple parameters, the counts in the rows labeled *Any* are smaller than the sum of the counts in the individual category or parameter rows below. *BSA* body surface area, *dp* difference in cardiac phase, *dSV* difference in ventricular stroke volumes, *LV* left ventricle, *LVEDV* left ventricular end diastolic volume, *LVEF* left ventricular ejection fraction, *LVESV* left ventricular end systolic volume, *RVEDV* right ventricular end diastolic volume, *RVEF* right ventricular ejection fraction, *RVESV* right ventricular end systolic volume, *SD* standard deviation

**Table 2:** Key characteristics and CMR metrics of the baseline dataset.

	Excluded	Included
	n = 2710	n = 26,899
Age, y	49.0 (13.0)	48.2 (12.2)
Women, %	35.6	45.1
Height, cm	174.9 (9.8)	173.0 (9.6)
Weight, kg	82.2 (17.9)	79.5 (16.2)
Diabetes, %	6.5	4.2
Hypertension, %	26.5	23.9
Myocardial Infarction, %	0.7	0.3
Dyslipidemia, %	22.1	22.3
ever smoking, %	52.7	49.6
Imaging site, %		
Site A	18	20.5
Site B	19.3	19.3
Site C	19.8	19.4
Site D	15.1	19.3
Site E	27.8	21.4
CMR metrics		
LVEDV, mL	143.9 (41.8)	142.0 (32.6)
LVESV, mL	63.9 (23.8)	52.3 (15.2)
LVSV, mL	80.0 (28.6)	89.7 (20.9)
LVEF, %	55.4 (11.1)	63.4 (5.3)
LV mass, g	122.6 (31.9)	113.4 (27.6)
RVEDV, mL	157.3 (46.4)	159.8 (38.5)
RVESV, mL	80.2 (28.9)	70.6 (21.9)
RVSV, mL	77.1 (28.2)	89.2 (22.4)
RVEF, %	49.0 (11.0)	56.1 (6.9)

Exclusions applied to participants who received scores of ≤3 in either image quality or segmentation quality (*primary* quality-controlled baseline dataset). Continuous data are provided as mean (SD). Categorical data are provided as percentages. Diabetes, hypertension, myocardial infarction, and dyslipidemia were ascertained by self-reported physician diagnoses. Ever-smoking was ascertained by self-report, considering current and former smoking. *CMR* cardiac magnetic resonance (imaging), *LV* left ventricle, *LVEDV* left ventricular end diastolic volume, *LVEF* left ventricular ejection fraction, *LVESV* left ventricular end systolic volume, *RVEDV* right ventricular end diastolic volume, *RVEF* right ventricular ejection fraction, *RVESV* right ventricular end systolic volume

Under the more permissive alternative approach, which also included participants rated as “3—moderate confidence,” exclusions decreased to 1196 of 5082 outliers (23.5%): 976 for image quality and 220 for segmentation issues. This resulted in an *auxiliary* baseline dataset comprising 28,413 of 29,908 participants (inclusion rate: 95.0%, Fig. 2).

**Fig. 4:**
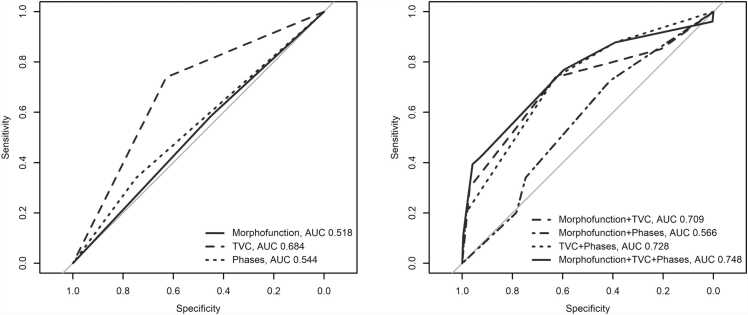
ROC and AUCs for outcome exclusion across outlier categories. The discriminative performance analysis draws on data from the 5082 participants identified as outliers. Exclusions applied to participants who received scores of ≤3 in either image quality or segmentation quality. The analysis considered single outlier categories (left) and their combinations (right). *AUC* areas under the curve, *ROC* receiver operating characteristics curves, *TVC* time-volume curves

Among the single outlier categories, time-volume curves (TVC) demonstrated the best discriminative ability to distinguish exclusion from inclusion, outperforming morphofunctionial criteria (difference in AUC: 0.166, 95%CI: [0.149, 0.182], p<0.001) and phase difference criteria (difference in AUC: 0.140 [0.119, 0.160], p<0.001). Among combinations of two categories, the combination of TVC and phase difference criteria showed the highest discriminative ability compared to the combination of morphofunctionial criteria and TVC (difference in AUC: 0.019 [0.010, 0.028], p<0.001) and the combination of morphofunctionial and phase difference criteria (difference in AUC: 0.162 [0.145, 0.179], p<0.001). Furthermore, the combination of TVC and phase difference criteria showed better discriminative ability than TVC alone (difference in AUC: 0.044 [0.039, 0.050], p<0.001). The combination of all three categories showed the best overall discriminative ability (difference in AUC to the combination of TVC and phase differences: 0.020 [0.014, 0.026], p<0.001) **(**Fig. 4**)**.

Proportions of outliers, image quality ratings, and exclusions by sex, age, and imaging site are provided in **Supplemental Table S7**. Exclusions based on image quality were more frequent in men than in women (10.6% vs 7.4%) and in older participants (>60 years) than in younger participants (30–60 years) (11.3% vs 8.3%), and reached a maximum of 11.6% at one imaging site.

### 3.2 Comparison of ventricular structure and function between included and excluded participants

**Fig. 5:**
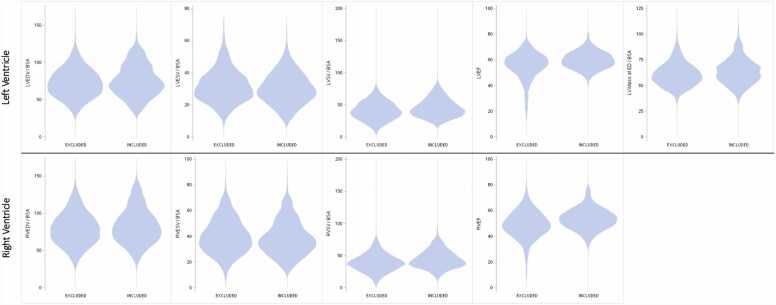
Violin plots illustrating the distributions of morphofunctional parameters among included and excluded participants within the outlier sample. The distributions are based on data from the 5082 participants identified as outliers. Exclusions applied to participants who received scores of ≤3 in either image quality or segmentation quality. *BSA* body surface area, *ED* end diastole, *LV* left ventricle, *LVEDV* left ventricular end diastolic volume, *LVEF* left ventricular ejection fraction, *LVESV* left ventricular end systolic volume, *LVSV* left ventricular stroke volume, *RVEDV* right ventricular end diastolic volume, *RVEF* right ventricular ejection fraction, *RVESV* right ventricular end systolic volume, *RVSV* right ventricular stroke volume

The distribution patterns of morphofunctional parameters for excluded and included outliers were similar in shape **(**Fig. 5**)**. Although the differences in median values were statistically significant, they were generally small: In excluded participants, decreases were observed in end-diastolic volumes (LVEDV: – 1.5 mL/m^2^, RVEDV: – 1.9 mL/m^2^, both p<0.001), stroke volumes (LVSV: – 3.6 mL/m^2^, RVSV: – 4.3 mL/m^2^, both p<0.001), ejection fractions (LVEF: – 2.3%, RVEF: – 4.3%, both p<0.001), and LV mass (– 3.5 g/m^2^, p<0.001), whereas increases were noted in end-systolic volumes (LVESV: + 1.0 ml/m^2^, RVESV: + 2.0 mL/m^2^, both p<0.001).

## 4 Discussion

This study on cardiac MR imaging data from the baseline sample of the NAKO study introduced a pipeline for automated segmentation of short-axis cine images coupled with a post-processing quality control workflow. The approach enabled the extraction of quantitative parameters and the exclusion of participants with suboptimal image or segmentation quality. As a result, we compiled a comprehensive data resource of cardiac structure and function from a large population-based cohort (n = 26,899 with complete or high diagnostic confidence and n = 28,413 with complete to moderate diagnostic confidence), providing a robust foundation for further analyses.

A challenge in segmenting large image datasets and performing quality control is the complex nature of potential interfering factors. Image quality can be degraded by a wide range of artifacts arising from the imaging equipment and its operation, from the examined individual’s conscious or unconscious movements, or from other, unrecognized factors affecting the imaging process. Similarly, segmentation quality may be compromised by various causes, including suboptimal input data or intrinsic limitations of the segmentation model. Identifying all relevant confounders requires considerable effort and may not be entirely feasible, however, detecting and excluding low-quality acquisitions is paramount to preserving the integrity of downstream analyses. In our study, we addressed this challenge by processing unfiltered short-axis cine images using a segmentation model based on the nnU-Net architecture, which had previously been validated to produce accurate and robust predictions [5], [6]. Building on the rationale that significantly impaired images would compromise segmentation quality or, if accurately segmented, yield implausible cardiac morphofunction parameters, we positioned statistical and visual quality control after segmentation, as opposed to prefiltering participants based on subpar short-axis cine images. This approach enabled us to identify participants with either impaired image quality, segmentation quality, or both. Our findings underscore the importance of expert visual quality controls alongside statistical methods in cardiac imaging, as exclusions could not be reliably determined by statistical criteria alone. This point is further illustrated by the similar parameter distribution shapes between included and excluded outliers **(**Fig. 5**)**. It should be noted, however, that these similarities may have been reinforced because quality constraints observed in the left ventricle did not necessarily coincide with limitations in the right ventricle, and vice versa, thereby contributing unremarkable parameter values to the distributions. A relatively strict penalization of inaccuracies by the rating scale may also have been a factor. Consequently, the median differences of morphofunctional parameters were generally small yet sufficiently consistent to reach statistical significance. When stratified by age and sex, exclusion frequency due to image quality was higher in male than female participants, and was elevated in the youngest and oldest age groups. The observed differences may be attributable to factors such as posture, bulk motion, breathing patterns, or compliance with instructions. However, these explanations remain speculative. Differences across imaging sites cannot be attributed to technical variation, but may instead reflect staff handling of protocol repetitions [14], [21] or participant heterogeneity. Although the final inclusion rate of 89.9% in the *primary* quality-controlled dataset may appear relatively low, it again reflects the application of strict quality standards—arguably more stringent than those commonly used in clinical practice. Another consideration is that in the NAKO study, CMR sequences were acquired towards the end of a nearly hour-long examination, potentially increasing the likelihood of lower-quality image data. Nonetheless, this proportion exceeds those reported in other large-scale CMR studies, where quality control assessments of samples from the UK Biobank and the Healthy Hearts Consortium resulted in inclusion rates above 95% for ventricular measurements [1], [22], [23]. For analyses that require less stringent quality control, the *auxiliary* dataset, with a higher inclusion rate of 95.0%, may provide a feasible alternative.

Other studies have explored deeper preprocessing methods for image quality control. In the UK Biobank, technically demanding approaches were employed to detect issues such as spatial or temporal inconsistencies between slices, primarily associated with variable breath-hold positions or inadequate ECG gating [24], [25], [26]. Techniques involving k-space reconstruction have also been utilized to detect and correct cine images from the UK Biobank dataset and an external cohort of healthy subjects [27], [28], [29]. Adapting and validating such algorithms for new datasets can require substantial effort. If only single slices in SAX image stacks are affected, a simpler alternative could involve omitting the affected slices followed by data interpolation from adjacent slices, thus limiting the introduced error and enabling reinclusion into a quality-controlled dataset. Preprocessing image quality control based on metrics like image sharpness, signal-to-noise ratio, and other general quantitative indicators has only shown moderate performance when applied to cardiac cine images from the NAKO [14], [21]. Separately, in the context of CMR image annotation and post-processing quality control, a multi-network approach was used for automatic segmentation alongside uncertainty estimation to detect segmentation failures [30]. Finally, an automated approach for expanding our quality scoring to the entire baseline dataset would, given the multiple factors affecting image and segmentation quality and heterogeneity of resulting artifact types, likely require a complex strategy involving multiple task-specific neural networks.

Our study focused not on shape-model accuracy, but on morphofunctional analysis, particularly in the context of end-diastolic (ED) and end-systolic (ES) phases. As a result, our methodology was not optimized to produce anatomically ideal segmentations, and interpolation artifacts were tolerated as long as they remained within the space of plausible annotations. While such artifacts may appear unfamiliar to the human eye, they do not fundamentally compromise the extraction of cardiac morphofunctional parameters. This issue is particularly relevant for the basal slices, where distinguishing atrial from ventricular borders is inherently challenging in short-axis images. This ambiguity complicates the differentiation between minor segmentation inaccuracies and true algorithmic failures. While such inaccuracies may average out at the whole-sample level, caution is warranted when interpreting individual participant data, even when segmentations received high-quality ratings. On this point, retraining the segmentation model is unlikely to yield substantial improvements unless long-axis cine images are incorporated to better delineate the atrioventricular borders.

## 5 Limitations

Our study has additional limitations. Most notably, we did not assess segmentation quality independently of image quality. This decision was based on several considerations. First, evaluating segmentation quality in isolation is inherently difficult, as it is closely dependent on the underlying image. For instance, minor motion artifacts due to ECG mistriggering or breathing may result in apparent segmentation deficiencies. Second, given our primary objective was to determine quality-controlled data on ventricular structure and function, segmentation quality alone was not a determining factor. Even so, the current approach still allows for the identification of participants potentially suitable for model retraining, specifically those with adequate image quality but insufficient segmentation quality scores. Regarding our method development, testing of inter-operator variability was limited to 30 participants and focused on parameters derived from the end-diastolic and end-systolic phases. Nonetheless, the segmentation model was extensively validated as part of the M&Ms challenge [5], [6]. While this does not entirely replace the need for inter-operator variability testing on the NAKO dataset, it provides supporting evidence of the overall accuracy and robustness of the segmentation model. The statistical outlier analysis also relied substantially on the end-diastolic and end-systolic phases and may therefore have failed to detect flaws that predominantly affect the intermediate phases. It also relied on sex-specific, non-age-stratified thresholds for stroke volume outlier detection. However, recent findings from the Healthy Hearts Consortium indicate that the relative influence of sex versus age varies with the CMR metric under consideration [31]. Moreover, the NAKO baseline cohort includes participants with cardiometabolic risk factors, prior CVD, and other diseases, which overall complicates the selection of appropriate criteria for defining outliers. Nonetheless, we are confident that our sex-stratified approach has identified the most relevant cases. An additional consideration in the context of cardiac phases is that identifying end-diastole and end-systole based on maximal and minimal ventricular volumes, as done in the present study, is an approximation. It is a practical compromise especially for the automated processing of large datasets such as the NAKO, and will have only a minor impact on the extracted global parameters. For analyses focusing on specific pathologies, however, a more precise definition of cardiac phases may be warranted. Furthermore, our methodology did not include a connected component analysis on the predicted segmentations. While this is unlikely to significantly impact morphofunctional parameter accuracy, such analysis would be necessary before reliably performing shape-model analyses on the segmentation data. Lastly, the validity of the LV parameters extracted in the present study is expected to exceed that of the RV parameters. Accurate segmentation of the RV remains challenging, particularly in basal planes where its borders to the right atrium are difficult to delineate. The segmentation model used in the present study frequently produced interpolations in these regions of uncertainty. As this was a known behavior of the model, such findings were classified as segmentation artifacts rather than true segmentation errors in the quality control process. While the inclusion of long-axis images can help to visualize the atrioventricular border more clearly, this does not necessarily improve segmentation accuracy, as demonstrated in the M&Ms-2 challenge [32]. In other studies, MR-based RV measurements were found to be more accurate when obtained from cine images in the body axial (transaxial) orientation, as opposed to the conventional cardiac short-axis view [33], [34].

## 6 Conclusion

The presented pipeline enabled automated segmentation of short-axis cine cardiac MR images from the baseline cohort of the NAKO study. By incorporating a thorough quality control process, we generated a comprehensive and reliable data resource of quantitative information on cardiac structure and function that is suitable for downstream analyses.

## Funding

P.M.F. was funded by the Kaltenbach Scholarship of the German Heart Foundation.

## Author contributions

**Peter M. Full:** Writing – original draft, Software, Methodology, Investigation, Formal analysis, Data curation, Conceptualization. **Robin T. Schirrmeister:** Writing – review & editing, Visualization, Methodology. **Manuel Hein:** Writing – review & editing, Methodology, Investigation. **Maximilian F. Russe:** Writing – review & editing, Software, Resources. **Marco Reisert:** Writing – review & editing, Software, Resources. **Clemens Ammann:** Writing – review & editing, Methodology, Investigation. **Karin Halina Greiser:** Writing – review & editing. **Thoralf Niendorf:** Writing – review & editing. **Tobias Pischon:** Writing – review & editing. **Jeanette Schulz-Menger:** Writing – review & editing, Validation, Methodology, Investigation. **Klaus H. Maier-Hein:** Writing – review & editing, Supervision, Software, Resources, Methodology. **Fabian Bamberg:** Writing – review & editing, Resources. **Susanne Rospleszcz:** Writing – original draft, Visualization, Validation, Project administration, Methodology, Investigation, Formal analysis. **Christopher L. Schlett:** Writing – review & editing, Validation, Supervision, Resources, Project administration, Methodology, Investigation, Conceptualization. **Christopher Schuppert:** Writing – original draft, Visualization, Validation, Supervision, Project administration, Methodology, Investigation, Formal analysis, Data curation, Conceptualization.

## Declaration of competing interests

The authors declare the following financial interests/personal relationships which may be considered as potential competing interests. Fabian Bamberg reports a relationship with Siemens Healthineers that includes: funding grants and speaking and lecture fees. Fabian Bamberg reports a relationship with Bayer Healthcare that includes: speaking and lecture fees. Jeanette Schulz-Menger reports a relationship with Siemens Healthineers that includes: funding grants. Christopher L. Schlett reports a relationship with Siemens Healthineers that includes: speaking and lecture fees. Christopher L. Schlett reports a relationship with Bayer Healthcare that includes: speaking and lecture fees. Other authors declare that they have no known competing financial interests or personal relationships that could have appeared to influence the work reported in this paper.

## Data Availability

Applications for access to NAKO data can be submitted via the NAKO web portal, *TransferHub* (https://transfer.nako.de). Each application must include, among other elements, a scientific justification for data use as well as a detailed description of the criteria and configurations under which the data will be utilized. All submissions are reviewed by the Use and Access Committee (UAC) within a four-week period, with consideration given to the scientific objectives and the potential benefits of the proposed research. When appropriate—particularly for applications involving specialized topics—additional experts may be consulted. These may include, for example, MRI module coordinators, representatives of the Competence Network for Secondary and Registry Data, or members of the biosample panel. If the UAC recommends revision, the applicant is automatically notified and invited to submit a revised application. If the UAC recommends approval, an automated notice is issued to the association members (i.e., stakeholders nominated by NAKO e.V. who are entitled to raise objections), initiating a four-week objection period. In the case of a rejected recommendation, the applicant is informed and given the option to withdraw the application and, if necessary, submit a new application. After the objection period, the Board of Directors typically makes a final decision within 2 weeks. The Board generally follows the UAC’s recommendation. If the application is approved, the principal applicant is automatically notified by email, and preparations begin for concluding the agreement with the main applicant’s affiliated institution. With regard to the data generated in the present study, the segmentation masks, the extracted cardiac morphofunctional parameters, as well as the quality control metrics will be available for use. For further details, please refer to the document *TFS-Info-03_EN_Information on Use and Access Procedure*, available at: https://transfer.nako.de/transfer/media/. The pretrained segmentation model used in this study has been previously published [6] and is publicly available at: https://doi.org/10.5281/zenodo.4134720 [16].

## References

[1] Petersen S.E., Aung N., Sanghvi M.M., Zemrak F., Fung K., Paiva J.M. (2017). Reference ranges for cardiac structure and function using cardiovascular magnetic resonance (CMR) in Caucasians from the UK Biobank population cohort. J Cardiovasc Magn Reson J Soc Cardiovasc Magn Reson.

[2] Petersen S.E., Khanji M.Y., Plein S., Lancellotti P., Bucciarelli-Ducci C. (2019). European Association of Cardiovascular Imaging expert consensus paper: a comprehensive review of cardiovascular magnetic resonance normal values of cardiac chamber size and aortic root in adults and recommendations for grading severity. Eur Heart J Cardiovasc Imaging.

[3] Isensee F., Jaeger P.F., Full P.M., Wolf I., Engelhardt S., Maier-Hein K.H. (2017). In: Conference Automatic Cardiac Disease Assessment on cine-MRI via Time-Series Segmentation and Domain Specific Features.

[4] Bernard O., Lalande A., Zotti C., Cervenansky F., Yang X., Heng P.A. (2018). Deep learning techniques for automatic MRI cardiac multi-structures segmentation and diagnosis: is the problem solved?. IEEE Trans Med Imaging.

[5] Campello V.M., Gkontra P., Izquierdo C., Martin-Isla C., Sojoudi A., Full P.M. (2021). Multi-centre, multi-vendor and multi-disease cardiac segmentation: the M&Ms challenge. IEEE Trans Med Imaging.

[6] Full P.M., Isensee F., Jäger P.F., Maier-Hein K. (2021). In: Conference Studying Robustness of Semantic Segmentation Under Domain Shift in Cardiac MRI.

[7] Attar R., Pereañez M., Gooya A., Albà X., Zhang L., de Vila M.H. (2019). Quantitative CMR population imaging on 20,000 subjects of the UK Biobank imaging study: LV/RV quantification pipeline and its evaluation. Med Image Anal.

[8] Bai W., Sinclair M., Tarroni G., Oktay O., Rajchl M., Vaillant G. (2018). Automated cardiovascular magnetic resonance image analysis with fully convolutional networks. J Cardiovasc Magn Reson J Soc Cardiovasc Magn Reson.

[9] Raisi-Estabragh Z., Petersen S.E. (2020). Cardiovascular research highlights from the UK Biobank: opportunities and challenges. Cardiovasc Res.

[10] Raisi-Estabragh Z., Harvey N.C., Neubauer S., Petersen S.E. (2021). Cardiovascular magnetic resonance imaging in the UK Biobank: a major international health research resource. Eur Heart J Cardiovasc Imaging.

[11] Peters A., German National Cohort C., Peters A., Greiser K.H., Gottlicher S., Ahrens W. (2022). Framework and baseline examination of the German National Cohort (NAKO). Eur J Epidemiol.

[12] Bamberg F., Schlett C.L., Caspers S., Ringhof S., Gunther M., Hirsch J.G. (2024). Baseline MRI examination in the NAKO health study-findings on feasibility, participation and dropout rates, comfort, and image quality. Dtsch Arztebl Int.

[13] Bamberg F., Kauczor H.U., Weckbach S., Schlett C.L., Forsting M., Ladd S.C. (2015). Whole-body MR imaging in the German National Cohort: rationale, design, and technical background. Radiology.

[14] Schuppert C., Kruchten R.V., Hirsch J.G., Rospleszcz S., Hoinkiss D.C., Selder S. (2022). Whole-body magnetic resonance imaging in the large population-based german national cohort study: predictive capability of automated image quality assessment for protocol repetitions. Invest Radio.

[15] Li X., Morgan P.S., Ashburner J., Smith J., Rorden C. (2016). The first step for neuroimaging data analysis: DICOM to NIfTI conversion. J Neurosci Methods.

[16] P.M. Full, F. Isensee, P.F. Jäger, K.H. Maier-Hein. Pretrained nnUNet Model cMRI M&Ms Challenge. 2020. doi: 10.5281/zenodo.4134720 (date last accessed).

[17] Schulz-Menger J., Bluemke D.A., Bremerich J., Flamm S.D., Fogel M.A., Friedrich M.G. (2013). Standardized image interpretation and post processing in cardiovascular magnetic resonance: Society for Cardiovascular Magnetic Resonance (SCMR) board of trustees task force on standardized post processing. J Cardiovasc Magn Reson J Soc Cardiovasc Magn Reson.

[18] Cerqueira M.D., Weissman N.J., Dilsizian V., Jacobs A.K., Kaul S., Laskey W.K. (2002). Standardized myocardial segmentation and nomenclature for tomographic imaging of the heart. A statement for healthcare professionals from the Cardiac Imaging Committee of the Council on Clinical Cardiology of the American Heart Association. Circulation.

[19] Sofroniew N., Lambert T., Bokota G., Nunez-Iglesias J., Sobolewski P., Sweet A. *et al. napari: a multi-dimensional image viewer for Python.* 10.5281/zenodo.16883660 (date last accessed).

[20] *The NORA Medical Imaging Platform Project.* 〈https://www.nora-imaging.org〉 (date last accessed).

[21] Schuppert C., Rospleszcz S., Hirsch J.G., Hoinkiss D.C., Köhn A., von Krüchten R. (2023). Automated image quality assessment for selecting among multiple magnetic resonance image acquisitions in the German National Cohort study. Sci Rep.

[22] Petersen S.E., Sanghvi M.M., Aung N., Cooper J.A., Paiva J.M., Zemrak F. (2017). The impact of cardiovascular risk factors on cardiac structure and function: insights from the UK Biobank imaging enhancement study. PLoS One.

[23] Chadalavada S., Rauseo E., Salih A., Naderi H., Khanji M., Vargas J.D. (2024). Quality control of cardiac magnetic resonance imaging segmentation, feature tracking, aortic flow, and native T1 analysis using automated batch processing in the UK Biobank study. Eur Heart J Imaging Methods Pr.

[24] Tarroni G., Oktay O., Bai W., Schuh A., Suzuki H., Passerat-Palmbach J. (2019). Learning-based quality control for cardiac MR images. IEEE Trans Med Imaging.

[25] Tarroni G., Bai W., Oktay O., Schuh A., Suzuki H., Glocker B. (2020). Large-scale quality control of cardiac imaging in population studies: application to UK biobank. Sci Rep.

[26] Robinson R., Valindria V.V., Bai W., Oktay O., Kainz B., Suzuki H. (2019). Automated quality control in image segmentation: application to the UK Biobank cardiovascular magnetic resonance imaging study. J Cardiovasc Magn Reson J Soc Cardiovasc Magn Reson.

[27] Oksuz I., Ruijsink B., Puyol-Antón E., Clough J.R., Cruz G., Bustin A. (2019). Automatic CNN-based detection of cardiac MR motion artefacts using k-space data augmentation and curriculum learning. Med Image Anal.

[28] Oksuz I., Clough J.R., Ruijsink B., Anton E.P., Bustin A., Cruz G. (2020). Deep learning-based detection and correction of cardiac MR motion artefacts during reconstruction for high-quality segmentation. IEEE Trans Med Imaging.

[29] Machado I., Puyol-Anton E., Hammernik K., Cruz G., Ugurlu D., Olakorede I. (2024). A deep learning-based integrated framework for quality-aware undersampled cine cardiac MRI reconstruction and analysis. IEEE Trans Biomed Eng.

[30] Sander J., de Vos B.D., Isgum I. (2020). Automatic segmentation with detection of local segmentation failures in cardiac MRI. Sci Rep.

[31] Raisi-Estabragh Z., Szabo L., McCracken C., Bulow R., Aquaro G.D., Andre F. (2024). Cardiovascular magnetic resonance reference ranges from the healthy hearts consortium. JACC Cardiovasc Imaging.

[32] Martin-Isla C., Campello V.M., Izquierdo C., Kushibar K., Sendra-Balcells C., Gkontra P. (2023). Deep learning segmentation of the right ventricle in cardiac MRI: the M&Ms challenge. IEEE J Biomed Health Inf.

[33] Alfakih K., Plein S., Bloomer T., Jones T., Ridgway J., Sivananthan M. (2003). Comparison of right ventricular volume measurements between axial and short axis orientation using steady-state free precession magnetic resonance imaging. J Magn Reson Imaging.

[34] Atalay M.K., Chang K.J., Grand D.J., Haji-Momenian S., Machan J.T., Sheehan F.H. (2013). The transaxial orientation is superior to both the short axis and horizontal long axis orientations for determining right ventricular volume and ejection fraction using Simpson's method with cardiac magnetic resonance. ISRN Cardiol.

