# Supplementary material for: Cardiac magnetic resonance imaging in the German National Cohort (NAKO): Automated segmentation of short-axis cine images and post-processing quality control
Source: J Cardiovasc Magn Reson. 2025 Sep 12;28(1):101958. doi: 10.1016/j.jocmr.2025.101958 (PMC13265423; doi:10.1016/j.jocmr.2025.101958)
Supplement: Supplementary file 1 — Supplementary material [file mmc1.docx]

**Supplementary Material**

**Supplemental Table S1.** Dice similarity coefficients between segmentations from an expert annotator using cvi42 and those generated by the nnU-Net segmentation algorithm

| **Phase** | **Segmentation** | **Dice Similarity Coefficient** | |
| --- | --- | --- | --- |
|  |  | **Mean** | **SD** |
| end-diastole | LV cavity | 0.95 | 0.02 |
|  | LV myocardium | 0.87 | 0.02 |
|  | RV cavity | 0.92 | 0.03 |
| end-systole | LV cavity | 0.88 | 0.05 |
|  | LV myocardium | 0.90 | 0.02 |
|  | RV cavity | 0.88 | 0.04 |

The analysis was limited to a single expert annotator due to technical unavailability of segmentation exports from the second expert (using syngo.via).
*SD, standard deviation; LV, left ventricle; RV, right ventricle*

**Supplemental Figure S2.** Comparison of morphofunctional parameters obtained from the nnU-Net segmentation algorithm and expert assessments performed by two experienced annotators in two software environments (syngo.via and cvi42) for a random sample of 30 participants – the results demonstrate that **(a)** differences, illustrated by Bland-Altman plots, and **(b)** correlations, shown in scatter plots, between results from the segmentation algorithm (labeled ‘nnU-Net’ below) and either expert assessment (labeled ‘Expert’ below) fall within the range of inter-reader variability observed between the two expert assessments

**Supplemental Figure S2.** (continued)


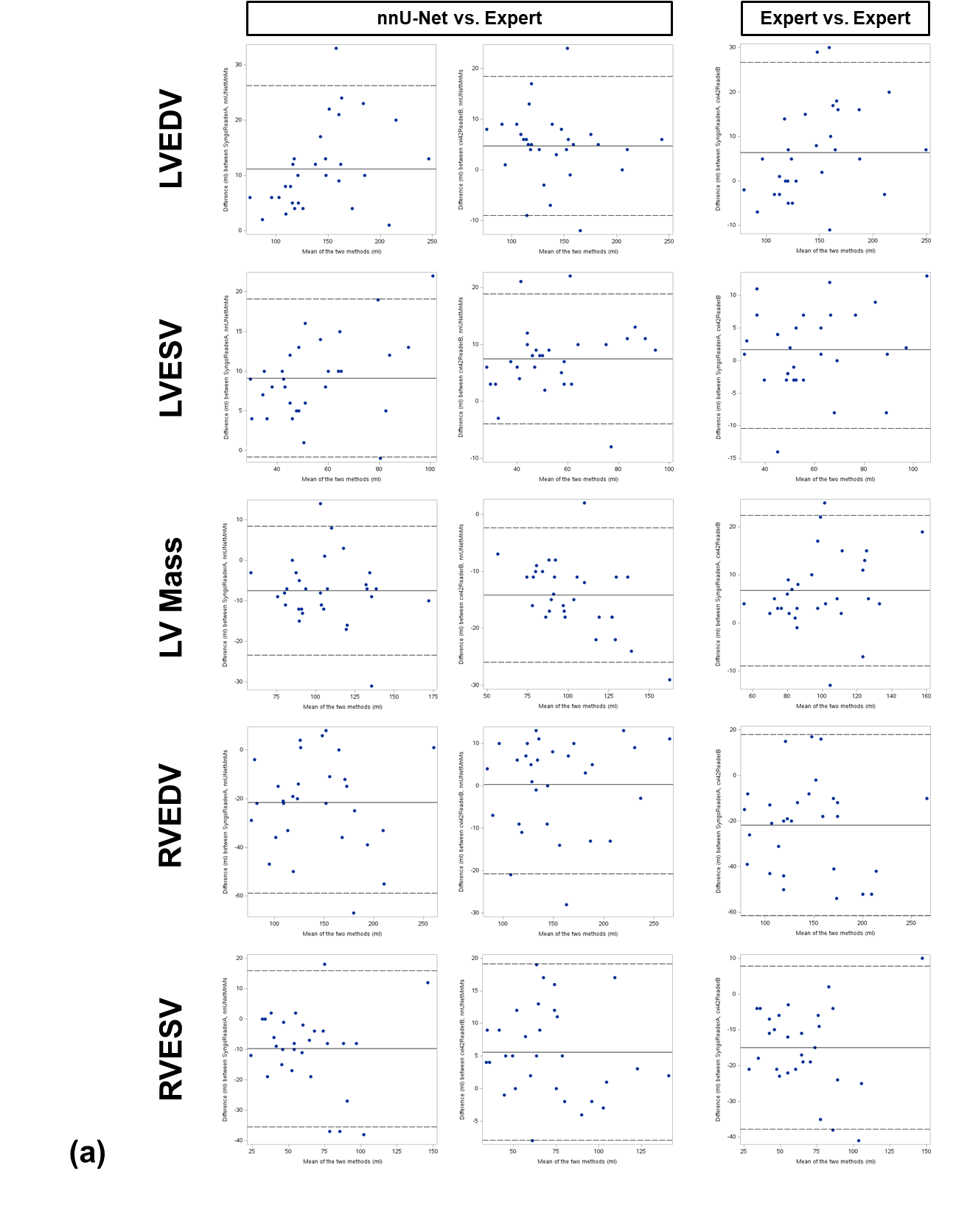
**Supplemental Figure S2.** (continued)


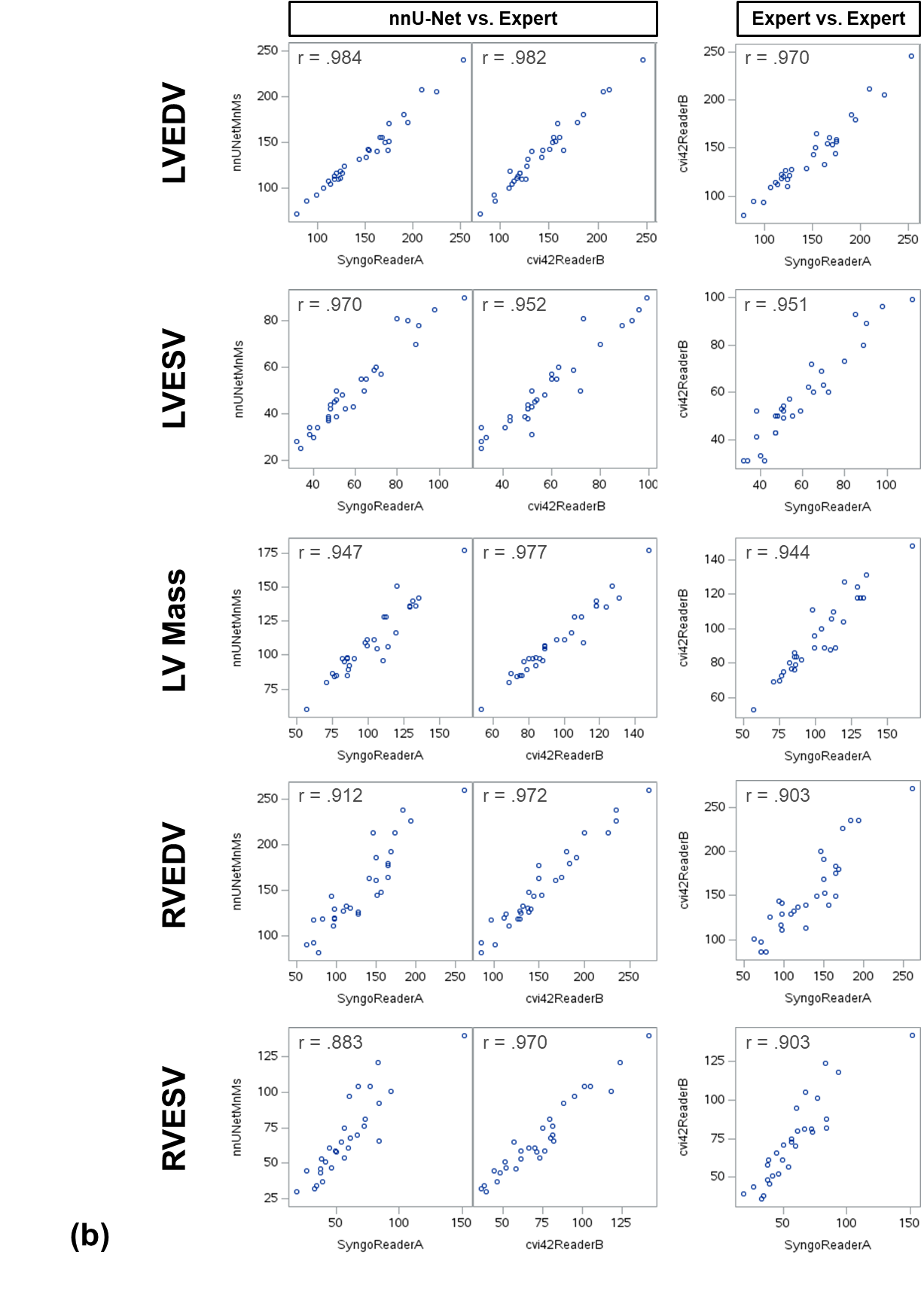


*LVEDV, left ventricular end diastolic volume; LVESV, left ventricular end systolic volume; RVEDV, right ventricular end diastolic volume; RVESV, right ventricular end systolic volume; r, Pearson correlation coefficient*

**Computation of Myocardial Wall Thickness**

A 17-segment AHA model for myocardial wall thickness at end-diastole was derived from the segmentation data. A schematic overview of the computation is shown in **Supplemental Figure S3**, with individual steps detailed below:

1. The volume of the LV mask was computed for all time steps and the time step with the largest volume was selected for further analysis.
2. The short-axis slices in the selected volume that contained any LV mask and any myocardial mask were divided equally into apical, mid-cavity, and basal thirds. This division could result in fractional assignments. For example, with ten slices, slices 1-3 are classified as 100 % apical, slice 4 as 33 % apical and 67 % mid-cavity, slices 5-6 as 100 % mid-cavity, slice 7 as 67 % mid-cavity and 33 % basal, and slices 8-10 as 100 % basal, such that each region (apical, mid-cavity, and basal) encompasses exactly 3⅓ slices.
3. For each such slice, endocardial and epicardial contour points, collectively forming the myocardial contour points, were identified: First, the contours of the myocardial mask were computed using the ‘marching squares’ method in scikit-image^[[1]](#footnote-1)^. Second, the myocardial center was estimated as the average of the myocardial point coordinates. Third, points were classified as inner or outer based on angle criteria: If the angle between the normal of the contour at a given point and the vector from the center to that point exceeded 90°, the point was considered an inner point, otherwise an outer point.
4. Myocardial thickness per outer point was calculated as the distance to the closest inner point.
5. The outer myocardial points were assigned to the corresponding myocardial segments. For that, the angles of all the vectors from the aforementioned myocardial center to the outer points were computed as well as the touching points between the myocardium and the RV (to determine the septum). The outer myocardial points belonging to the septum were further subdivided into the corresponding two septal segments in the basal and mid-cavity slices. The remaining outer points were subdivided into three (apical) or five (basal, mid-cavity) segments as per the AHA definition^[[2]](#footnote-2)^. All subdivisions were based on the aforementioned angles. Slices in which more than 50% of the myocardial circumference was absent at end diastole—typically occurring at the left ventricular outflow tract—were excluded, whereas slices retaining more than 50% of the circumferential coverage were retained. However, to avoid bias in wall thickness estimation, 4.5 mm on either side of the ‘gaps’ (i.e., the outflow tract) were removed.
6. The segment thickness was then computed as the average of the myocardial thicknesses in that segment, also taking into account the fractional assignments of slices to apical, mid-cavity and basal as explained in step 2.

**Supplemental Figure S3.** Schematic overview of the myocardial wall thickness computation.


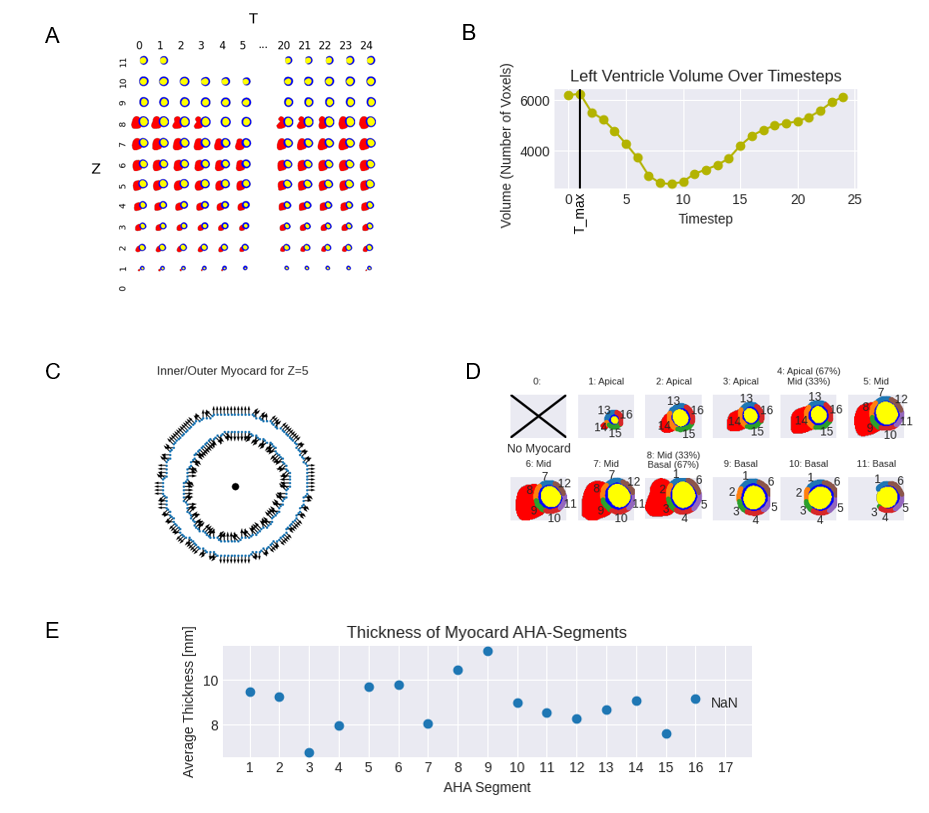


**A:** Segmentations across all phases and slices as input. **B:** Selection of the phase with maximum left ventricular volume. **C:** Identification of endocardial and epicardial contour points. **D:** Assignment of points to AHA segments. **E:** Computation of myocardial thickness per AHA segment as final output.

**Supplemental Figure S4.** The rating scales employed for the visual evaluation of the cardiac short-axis cine images during the quality control reads

**Supplemental Figure S4.** (continued)

*The image quality rating was performed first and especially considered included motion artifacts from breathing or inconsistent ECG-synchronization (in this context, ‘asynchronous slices’ refers to temporal inconsistencies in the short-axis image stack), misalignment from the intended short-axis orientation, and missing slices. The subsequent segmentation quality rating addressed errors such as oversegmentation or undersegmentation, while allowing artifacts inherently associated with the segmentation method and its handling of interpolation or partial-volume effects. The combined rating could only be as high as the initial image quality rating.*

**Supplemental Figure S5.** The image displays the NORA image viewer and the layout selected for visually evaluating cardiac short-axis cine images during the quality control reads


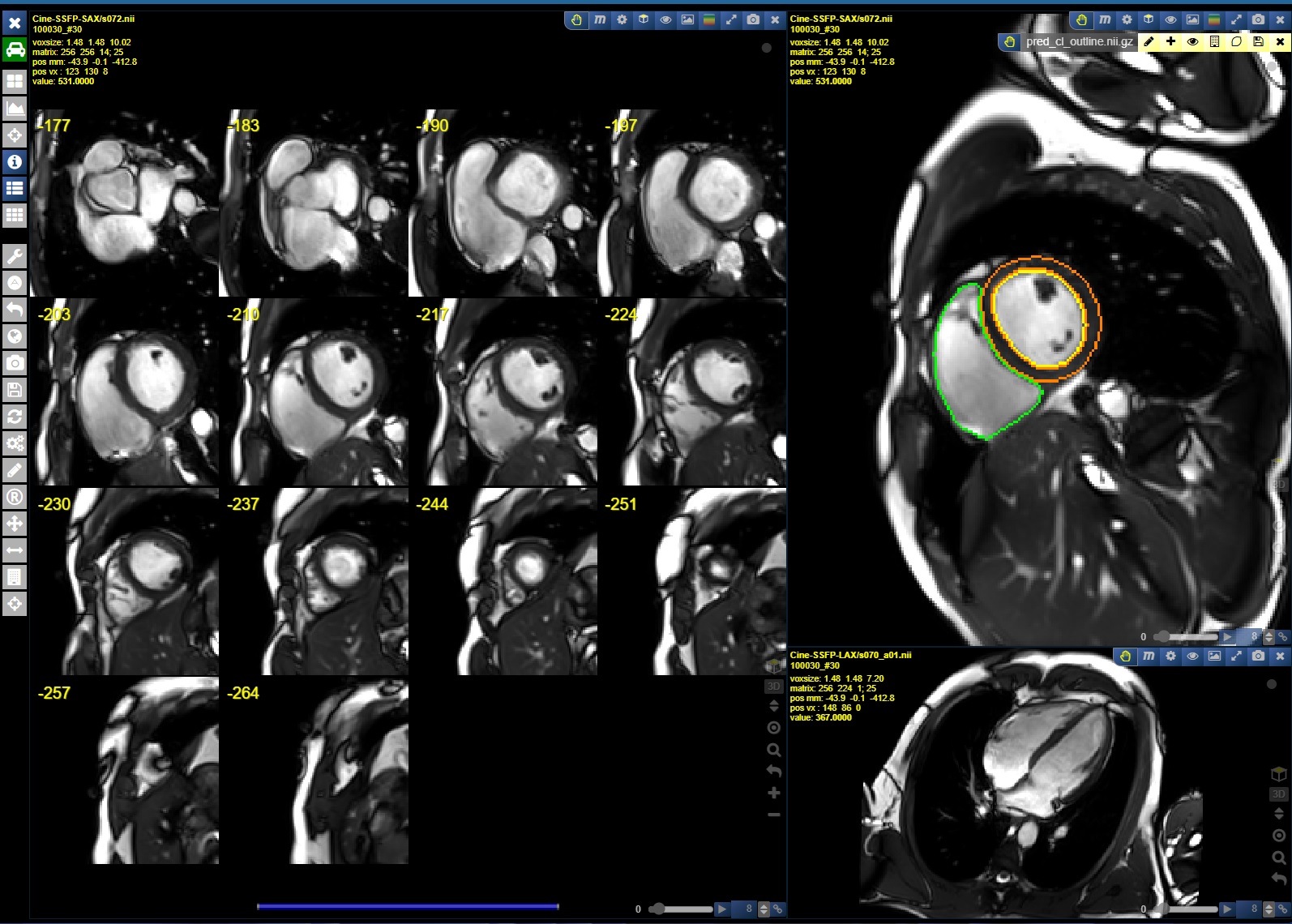


**Supplemental Figure S6.** Representative examples of quality issues that, depending on severity, prevented inclusion into the quality-controlled dataset

**(a)** shows the effect of ECG mistriggering, resulting in reduced cardiac sharpness. **(b)** Cardiac blurriness due to breathing, also affecting the diaphragm and spleen (white asterisk). **(c)**Banding artifact predominantly interfering with visualization of the left ventricle. **(d)** Extensive oversegmentation of the right ventricular contour (green) into the pericardial fat.
*Although the observed quality issues were heterogeneous and extended beyond those illustrated, ECG mistriggering and breathing artifacts were* particularly *common.*

**Supplemental Table S7.** Proportion of outliers, image quality ratings, and exclusions according to different stratifications

|  | **Sex** | |  | **Age** | | | | |  | **Imaging Site** | | | | |
| --- | --- | --- | --- | --- | --- | --- | --- | --- | --- | --- | --- | --- | --- | --- |
|  | **Men** | **Women** |  | **20-29** | **30-39** | **40-49** | **50-59** | **60+** |  | **Site A** | **Site B** | **Site C** | **Site D** | **Site E** |
| % of total sample | 55.8 | 44.2 |  | 10.4 | 11.9 | 29.4 | 27.7 | 20.6 |  | 20.3 | 19.3 | 19.4 | 18.9 | 22 |
| % outliers | 20 | 13.6 |  | 17.6 | 14.6 | 15.4 | 16.6 | 21.4 |  | 15.5 | 17 | 17.3 | 15.8 | 19.9 |
| Image Quality Rating, % |  |  |  |  |  |  |  |  |  |  |  |  |  |  |
| 1 | 2.2 | 1.3 |  | 1.4 | 1.3 | 1.5 | 1.8 | 2.8 |  | 1.7 | 1.6 | 1.5 | 0.7 | 3.3 |
| 2 | 1.9 | 0.9 |  | 1.3 | 0.9 | 1.1 | 1.6 | 2.3 |  | 1.1 | 1.4 | 1.6 | 1.1 | 2.1 |
| 3 | 3.9 | 1.9 |  | 2.4 | 2.5 | 2.7 | 3.1 | 4.2 |  | 2.4 | 3.1 | 3.2 | 2.6 | 3.8 |
| 4 | 8.3 | 6.2 |  | 9.3 | 7.2 | 6.8 | 6.7 | 8.3 |  | 6.9 | 7.6 | 7.6 | 7.5 | 7.2 |
| 5 | 3.7 | 3.2 |  | 3.2 | 2.8 | 3.4 | 3.4 | 4.2 |  | 3.4 | 3.3 | 3.4 | 3.9 | 3.4 |
| % excluded | 10.6 | 7.4 |  | 9.9 | 8.3 | 8.3 | 8.5 | 11.3 |  | 8.1 | 9.1 | 9.3 | 7.3 | 11.6 |

1. van der Walt S, Schönberger JL, Nunez-Iglesias J, Boulogne F, Warner JD, Yager N, et al. scikit-image: image processing in Python. PeerJ. 2014 2014/06/19;**2**:e453. [↑](#footnote-ref-1)
2. Cerqueira MD, Weissman NJ, Dilsizian V, Jacobs AK, Kaul S, Laskey WK, et al. Standardized myocardial segmentation and nomenclature for tomographic imaging of the heart. A statement for healthcare professionals from the Cardiac Imaging Committee of the Council on Clinical Cardiology of the American Heart Association. Circulation. 2002 Jan 29;**105**(4):539-42. [↑](#footnote-ref-2)
